# Rapid metabolic pathway assembly and modification using serine integrase site-specific recombination

**DOI:** 10.1093/nar/gkt1101

**Published:** 2013-11-12

**Authors:** Sean D. Colloms, Christine A. Merrick, Femi J. Olorunniji, W. Marshall Stark, Margaret C. M. Smith, Anne Osbourn, Jay D. Keasling, Susan J. Rosser

**Affiliations:** ^1^Institute of Molecular, Cell and Systems Biology, University of Glasgow, Bower Building, University Avenue, Glasgow G12 8QQ, Scotland, UK, ^2^Department of Biology, University of York, Wentworth Way, York YO10 5DD, UK, ^3^Department of Metabolic Biology, The John Innes Centre, Norwich Research Park, Norwich NR4 7UH, UK, ^4^Joint BioEnergy Institute, Emeryville, CA 94608, USA, ^5^Department of Chemical and Biomolecular Engineering, University of California, Berkeley, CA 94720, USA and ^6^Physical Biosciences Division, Lawrence Berkeley National Laboratory, Berkeley, CA 94720, USA

## Abstract

Synthetic biology requires effective methods to assemble DNA parts into devices and to modify these devices once made. Here we demonstrate a convenient rapid procedure for DNA fragment assembly using site-specific recombination by ϕC31 integrase. Using six orthogonal *attP*/*attB* recombination site pairs with different overlap sequences, we can assemble up to five DNA fragments in a defined order and insert them into a plasmid vector in a single recombination reaction. ϕC31 integrase-mediated assembly is highly efficient, allowing production of large libraries suitable for combinatorial gene assembly strategies. The resultant assemblies contain arrays of DNA cassettes separated by recombination sites, which can be used to manipulate the assembly by further recombination. We illustrate the utility of these procedures to (i) assemble functional metabolic pathways containing three, four or five genes; (ii) optimize productivity of two model metabolic pathways by combinatorial assembly with randomization of gene order or ribosome binding site strength; and (iii) modify an assembled metabolic pathway by gene replacement or addition.

## INTRODUCTION

Synthetic biology seeks to engineer new function in biological systems for a variety of different applications. A key limitation in synthetic biology is the time taken to assemble genes or other DNA parts into new devices, pathways and systems, and to alter these assemblies once made. The current methods for gene assembly and systematic variation of DNA parts are far from ideal (1). Existing assembly methods use restriction enzymes and DNA ligases to join DNA segments together, either sequentially (2,3) or in a single reaction (4,5), or use homology-dependent assembly *in vitro* (6–9) or *in vivo* (10–13). The junction sequences left by these methods make it difficult to change individual parts of the assembly without starting the assembly process from the beginning again. Furthermore, most of these methods cannot produce large libraries of variant assemblies from large numbers of DNA parts for use in combinatorial assembly strategies. Here we report the development of a highly efficient, rapid and easy recombinational assembly method (SIRA, serine integrase recombinational assembly) that uses ϕC31 integrase to assemble pre-designed pathways and devices from DNA parts. We illustrate the use of SIRA to assemble functional metabolic pathways from individual gene cassettes. Once assembled, genes or other DNA elements can be replaced, deleted or added at will. The assembly process can easily incorporate multiple variants at all gene positions, randomize gene order and vary expression levels, allowing rapid pathway optimization.

## MATERIALS AND METHODS

### Bacterial strains and growth

Routine bacterial growth was in LB medium (14), with agar (15 g/l) and antibiotics (ampicillin 100 µg/ml, chloramphenicol 25 µg/ml, kanamycin 50 µg/ml) added as required. *Escherichia coli* TOP10 (Life Technologies) was used for routine transformation and maintenance of plasmids. *E. coli* DB3.1 carrying the *gyrA462* mutation (15) was used for propagation of plasmids containing the *ccdB* gene. *E. coli* DS941-Z1, carrying the *lacI* and *tetR* genes from DH5αZ1 (16) P1 transduced into DS941 (17), was used for expression of the violacein biosynthetic pathway.

*E. coli* cells were made chemically competent with 50 mM calcium chloride and transformed using standard methods (14). Transformation routinely yielded between 2 × 10^6^ and 6 × 10^6^ transformants per µg pUC19 plasmid DNA. Electrocompetent TOP10 *E. coli* were prepared by repeated washes in ice-cold 10% glycerol (14) and electroporated using a Biorad micro pulser apparatus set on EC2 (2.5 kV, 5.9 ms time-constant), in a 2 mm cuvette according to the manufacturer’s instructions. Electroporation routinely yielded ∼1 × 10^8^ transformants per µg pUC19 plasmid DNA.

*Pantoea ananatis* (DSM number 30080; formerly *Erwinia uredovora*), containing the carotenoid biosynthetic gene cluster (Genbank accesssion number D90087) and *Chromobacterium violaceum* (DSM number 30191), containing the violacein biosynthetic gene cluster (Genbank accession number NC_005085 coordinates 3558964-3566288) were obtained from the DSMZ German collection of microorganisms and cell cultures. Genomic DNA was purified from these organisms using the QIAGEN Gentra Puregene kit according to the manufacturer’s instructions for Gram-negative bacteria.

### Plasmid construction

The plasmids used in this study are shown in Table 1. Oligonucleotides used in their constructions are shown in Supplementary Table S1.1. Biobricks were obtained from the registry of standard biological parts (http://parts.igem.org). Full plasmid sequences are available on request. pSIRA vectors were based on pSB1A3, an ampicillin-resistant pMB1 origin vector containing the P1010 *ccdB* biobrick (3). pSIRA1 was constructed by inserting *attB*-RX-TT top and bottom strands between EcoRI and XbaI sites of pSB1A3, and *attB*-PS-TC top and bottom strands between PstI and SpeI. pSIRA2 was constructed in the same way with *attP*-RX-TT and *attP*-PS-TC oligonucleotides, whereas pSIRA3 used *attB*-RX-TT and *attP*-SP-TC oligonucleotides. pL-lacO and pL-tetO promoters (16) were made by annealing pL-Eco top and bottom strands with either pL-tet or pL-lac top and bottom strands and inserted into the EcoRI site of pSIRA1 to produce pSIRA4 and pSIRA5, respectively. The *crtB*, *crtE*, *crtI* and *crtY* genes were amplified together with designed ribosome binding sites (RBS) by PCR using Phusion polymerase (New England Biolabs) and the primers shown in Supplementary Table S1.2 and inserted into pCR2.1-TOPO by TOPO-TA cloning (Life Technologies) to create pCM1, pCM2, pCM3 and pCM4, respectively. p*-ccdB*-Cm^R^ was constructed by inserting the P1004 chloramphenicol resistance biobrick as an XbaI-PstI fragment into SpeI-PstI-digested pSB1A3 already containing the P1010 *ccdB* biobrick.

**Table 1.** Plasmids used in this study

Plasmid name	Description	Resistance	Reference
pCR®2.1-TOPO	TOPO TA cloning vector	Amp^R^ Kan^R^	Life technologies
pSB1A3	pMB1 *ori* biobrick cloning vector containing the P1010 *ccdB* cell death biobrick	Amp^R^	Registry of biological parts (3)
pSW23	suicide cloning vector oriV(R6Kgamma)	Cm^R^	Demarre *et al.* (18)
pSW29	suicide cloning vector oriV(R6Kgamma)	Kan^R^	Demarre *et al.* (18)
pCM1	*P. ananatis crtB* PCR product in pCR®2.1-TOPO	Amp^R^ Kan^R^	This work
pCM2	*P. ananatis crtE* PCR product in pCR®2.1-TOPO	Amp^R^ Kan^R^	This work
pCM3	*P. ananatis crtI* PCR product in pCR®2.1-TOPO	Amp^R^ Kan^R^	This work
pCM4	*P. ananatis crtY* PCR product in pCR®2.1-TOPO	Amp^R^ Kan^R^	This work
p-*ccdB* -Cm^R^	pSB1A3 with P1010 (*ccdB*) and P1004 (Cm^R)^	Amp^R^ Cm^R^	This work
pSIRA1	*attB*-TT *attB*-TC SIRA vector based on pSB1A3	Amp^R^	This work
pSIRA2	*attP*-TT *attP*-TC SIRA vector based on pSB1A3	Amp^R^	This work
pSIRA3	*attB*-TT *attP*-TC SIRA vector based on pSB1A3	Amp^R^	This work
pSIRA4	pSIRA1 with pL-tet upstream of *attP*-TT	Amp^R^	This work
pSIRA5	pSIRA1 with pL-lac upstream of *attP*-TT	Amp^R^	This work

### PCR

Fragments for gene assembly were amplified by PCR using Phusion high-fidelity DNA polymerase (New England Biolabs) with the primers and templates shown in Supplementary Table S1.3. Reactions (50 µl) generally contained 1 × HF Phusion buffer with an additional 0.5 mM MgCl_2_, 200 µM of each deoxynucleotide triphosphate, 3% dimethyl sulfoxide, 500 nM of each primer, ∼1 ng of plasmid template or 50 ng genomic template and 0.5 µl Phusion polymerase. Reactions were subjected to an initial denaturation step of 98°C for 1 min, followed by 30 cycles of 98°C for 20 s, 55°C for 30 s and 72°C for 60–120 s. After a final step of 72°C for 10 min, products were separated on 1% agarose gels in tris-acetate buffer [40 mM Tris base, 20 mM acetic acid, 1 mM ethylenediaminetetraacetic acid (EDTA)], stained with ethidium bromide, excised under long-wave ultraviolet illumination and purified using a QIAquick gel extraction kit (QIAGEN) according to the manufacturer's instructions, with an extra wash step with 0.5 ml buffer PE. Purified PCR products were quantified by agarose gel electrophoresis with known DNA standards or by measuring UV absorbance at 260 nm.

### Proteins

The ϕC31 integrase and the *gp3*-encoded recombination directionality factor (RDF) were purified as described by Olorunniji *et al.*(19). Proteins were diluted in a buffer containing 25 mM Tris–HCl (pH 7.5), 1 mM DTT, 1 M NaCl and 50% (vol/vol) glycerol and stored at −20°C.

### Assembly reactions

For SIRA reactions, each PCR fragment (final concentration ∼5 nM) and vector (final concentration ∼3 nM) were mixed together in a 20 µl reaction containing 10 mM Tris–HCl (pH 7.5), 5 mM spermidine·3HCl, 2 mM dithiothreitol, 0.1 mM EDTA, 0.1 mg/ml bovine serum albumin and between 0 and 20% (vol/vol) ethylene glycol. Recombination was initiated by the addition of 2 µl of 2 µM ϕC31 integrase in protein dilution buffer. For *attL* × *attR* recombination, equal volumes of 4 µM integrase and 8 µM RDF were pre-mixed in protein dilution buffer and incubated on ice for 10 min, and reactions were started by adding 2 µl of this mixture. Reactions were incubated at 30°C for the indicated time (5 min–24 h) and then stopped by heating to 75°C for 10 min. For chemical transformation, 2 µl of heat-treated assembly reaction was added directly to 100 µl of competent *E. coli* cells. For electroporation, DNA was first precipitated by the addition of 2.2 volumes of 100% ethanol in the presence of 0.3 M sodium acetate and 1 µg of yeast tRNA (Sigma-Aldrich). Precipitated DNA was re-dissolved in 10 µl of distilled H_2_O, and 2 µl of this DNA was used to transform 30 µl of electrocompetent cells.

## RESULTS

### Recombinational cloning using ϕC31 integrase

**Figure 1.**
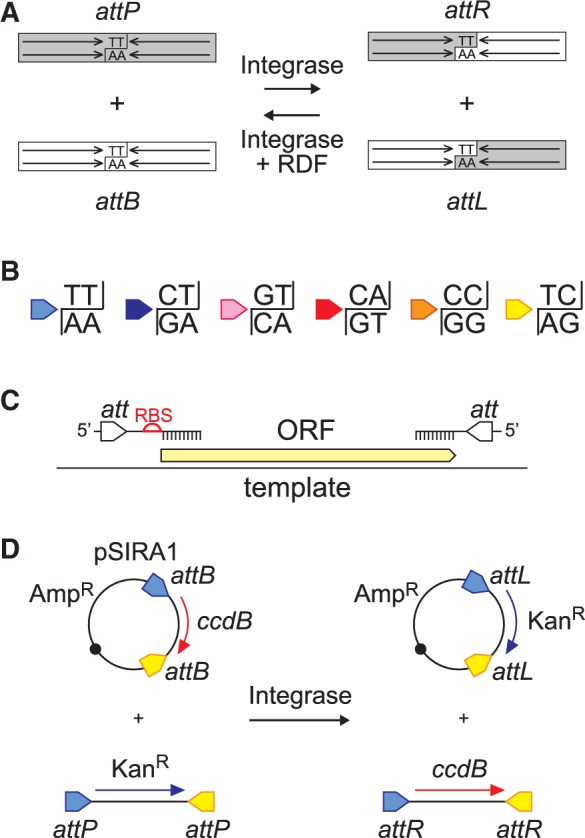
The SIRA process. (**A**) In the presence of ϕC31 integrase alone, *attP* and *attB* sites recombine to form *attL* and *attR*. The reaction can be reversed in the presence of the RDF. Integrase cuts both strands near the centres of the sites at positions staggered by two nucleotides. These central overlap sequences define the left–right polarity of the sites and must match in recombining sites. (**B**) Six possible asymmetric central overlap sequences can be used to produce pairs of orthogonal sites that will recombine only with each other and will be cut and rejoined in a specific orientation. The colour code shown here is used throughout the rest of the article. (**C**) RBS sequences and *att* sites can be incorporated into DNA fragments by PCR. Recombination sites are in inverted repeat to reduce complementarity between forward and reverse PCR primers for each cassette. (**D**) Inserting a single DNA fragment (here encoding a Kan^R^ determinant) into a vector by SIRA cassette exchange. *attP* sites at the ends of a linear DNA fragment recombine with matching *attB* sites in the pSIRA vector, replacing the conditionally lethal *ccdB* gene with the linear fragment. The desired product can be selected by its ability to transform a *ccdB*-sensitive *E. coli* strain.

Bacteriophage ϕC31 integrase inserts the phage genome into its host’s DNA by recombining specific ∼46 bp *attP* and *attB* DNA sequences found in the phage and host genomes, respectively (20,21). Integrase makes staggered cuts on either side of a central two base-pair ‘overlap’ sequence in both *attP* and *attB* (22), exchanges the resulting half-sites and rejoins them to form two new sites, *attL* and *attR* (Figure 1A). Recombination between *attP* and *attB* is highly efficient both *in vitro* and *in vivo*. In the presence of integrase alone, the products of recombination between *attP* and *attB* (*attL* and *attR*) do not recombine further, and the reaction is highly directional (23). However, the addition of the phage-encoded recombination directionality protein (RDF) allows efficient recombination between *attL* and *attR* and inhibits recombination between *attP* and *attB* (24).

The central 2 bp overlap sequences of two sites must match perfectly for efficient recombination, but their precise sequence has little effect on the efficiency of recombination (25,26). Throughout this article, we refer to *attP* with the 2 bp overlap sequence 5′-TT on the top strand (and 5′-AA on the bottom strand) as *attP*^TT^ and so forth. Thus, *attP*^TT^ recombines efficiently only with *attB*^TT^, *attP*^TC^ with *attB*^TC^ and so on. The 2 bp overlap sequence also determines the polarity of *attP* and *attB* sites, so that for instance *attP*^TT^ will recombine with a reversed *attB*^AA^ site, because the 5′-TT on the top strand of *attP*^TT^ matches the 5′-TT on the bottom strand of *attB*^AA^ (25). Furthermore, *att* sites with symmetrical central 2 bp overlap sequences (e.g. *attP*^TA^ and *attB*^TA^) will recombine in both possible relative orientations. Therefore, six different matched *attP*/*attB* pairs that recombine only with each other in a defined orientation can be made using the non-symmetric overlap sequences (top strand/bottom strand) 5′-TT/5′-AA, 5′-CT/5′-AG, 5′-GT/5′-AC, 5′-CA/5′-TG, 5′-CC/5′-GG and 5′-TC/5′-GA (Figure 1B, Supplementary Figure S1A). Because each *attP* site recombines with its matching *attB* site but not with any other, we refer to these matched *att* site pairs as ‘orthogonal’.

Using these six orthogonal recombination site pairs, we have developed a recombinational assembly and cloning strategy. This SIRA strategy uses plasmid vectors containing two *att* sites with different central dinucleotides, flanking a conditionally lethal *ccdB* gene. Recombination between the sites on the plasmid and matching sites at either end of a linear DNA fragment replaces *ccdB* with the linear fragment (Figure 1D). The product can easily be isolated by selection for loss of *ccdB*. We have generated a set of pSIRA vectors containing different *att* sites and regulated promoters to express the inserted genes (Table 1).

We first demonstrated that purified ϕC31 integrase can be used for efficient insertion of a single linear DNA fragment into a plasmid vector by cassette exchange. A kanamycin resistance gene flanked by *attP* sites with two different central dinucleotides (*attP*^TT^ and *attP*^TC^) was generated by PCR (Figure 1C) and mixed with pSIRA1, which carries *attB*^TT^ and *attB*^TC^ sites flanking *ccdB* (Figure 1D). After reacting for 1 h with ϕC31 integrase, the reaction products were electroporated into TOP10 *E. coli* cells and transformants were selected for resistance to ampicillin and then tested for resistance to kanamycin. All transformants tested (100/100) were resistant to kanamycin (Table 2; Supplementary Figure S2), confirming that the linear DNA fragment was efficiently exchanged into pSIRA1. This single-gene SIRA reaction routinely gave 8 × 10^7^ colonies per µg of vector DNA, corresponding to 9 × 10^6^ colonies from a single SIRA cloning reaction (Table 2). Analysis of plasmid DNA from a sample of colonies confirmed that they contained the kanamycin resistance gene from the PCR product in single copy with a defined orientation, as expected if each *attP* can recombine only with its matching *attB* site (data not shown). The reaction was efficient enough that calcium chloride transformation could be used instead of electroporation (Table 2), and a five-minute SIRA reaction was sufficient to clone a single DNA fragment (Supplementary Figure S2). Similar results were obtained with kanamycin or chloramphenicol resistance cassettes flanked by two *attB* sites or one *attP* and one *attB* site, with pSIRA2 and pSIRA3, respectively (Supplementary Figure S2). A small number of colonies lacking the expected antibiotic resistance cassette were detected with pSIRA3 (containing *attB*^TT^ and *attP*^TC^), suggesting a low level of intramolecular recombination between the mismatched *attP* and *attB* sites in the vector, leading to deletion of *ccdB* (Supplementary Figure S2).

**Table 2.** Efficiency of SIRA reactions

Number of fragments assembled	Pathway	Reaction time	Transformants per 20 µl reaction (CaCl_2_)^a^	Transformants per 20 µl reaction (electrocompetent)^b^	Transformants per µg vector DNA^c^	Percent correctly assembled (%)
1	Kan^R^	1 h	2 × 10^4^	9 × 10^6^	8 × 10^7^	100
3	*crtBEI*	4 h	4 × 10^3^	8 × 10^5^	7 × 10^6^	59
3	*crtBEI*	22 h	1 × 10^4^	1 × 10^6^	9 × 10^6^	87
4	*crtBEIY*	24 h	9 × 10^3^	1 × 10^6^	9 × 10^6^	37
5	*crtBEIZY*	22 h	9 × 10^3^	7 × 10^5^	6 × 10^6^	18
5	*vioABCDE*	22 h	2 × 10^3^	4 × 10^5^	4 × 10^6^	18
Replacement^d^	crtBEI*ZY	22 h	9 × 10^3^	4 × 10^5^	1 × 10^6^	94
Addition^e^	*crtI-idi-dxs*	16 h	7 × 10^2^	1 × 10^5^	3 × 10^5^	48

^a^Number of transformants obtained from a 20 µl assembly reaction introduced into CaCl_2_ TOP10 competent cells (∼10^6^ transformants per µg pUC19).

^b^Number of transformants obtained from a 20 µl assembly reaction introduced into electrocompetent TOP10 cells (∼10^8^ transformants per µg pUC19).

^c^Number of transformants obtained per µg of vector DNA using electrocompetent TOP10 cells (∼10^8^ transformants per µg pUC19).

^d^Replacement of the *crtI* gene in the 5-gene *crtBEIZY* assembly with a Phusion-amplified *crtI* cassette, after first replacing *crtI* with the *ccdB*-Cm^R^cassette.

^e^Replacement of the *crtI* gene in the 5-gene *crtBEIZY* assembly with *crtI, idi* and *dxs*, after first replacing *crtI* with the *ccdB*-Cm^R^ cassette.

### Metabolic pathway assembly by SIRA

**Figure 2.**
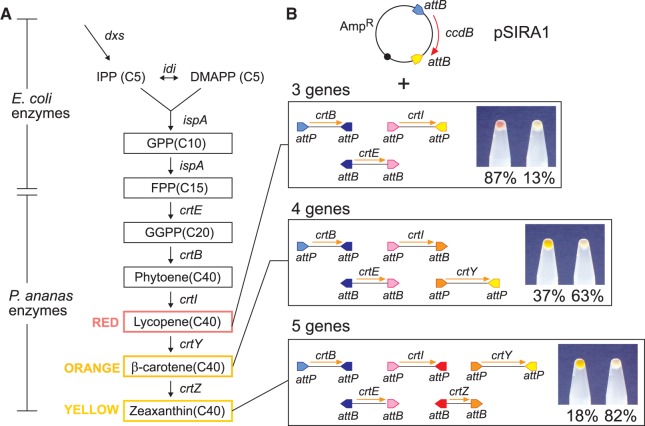
Assembly of 3-, 4- and 5-gene carotenoid biosynthetic pathways. (**A**) The *E. coli* host genes *idi* and *dxs* encode enzymes necessary for the production of IPP and DMAPP*.* IPP and DMAPP are converted to geranyl pyrophosphate (GPP) and farnesyl pyrophosphate (FPP) by further *E. coli* enzymes. The products of the *crtE, crtB*, *crtI*, *crtY* and *crtZ* genes from *P. ananas* convert FPP via geranylgeranyl pyrophosphate (GGPP) and phytoene to the coloured carotenoids lycopene (red), β-carotene (yellow–orange) and zeaxanthin (yellow). **(B)** Assembly of the three-gene lycopene, four-gene β-carotene and five-gene zeaxanthin biosynthetic pathways by SIRA. The diagrams show the arrangement of *att* sites used on each fragment for ordered assembly of genes into pSIRA1. The photographs show cell pellets from one correctly assembled construct (coloured) and one incorrectly assembled construct (white) for each pathway. The percentages of transformant colonies that were coloured or white are shown below each pellet. Cell pellets from 4- and 5-gene assemblies had similar colours, but restriction analysis confirmed that plasmids from these reactions all had the expected 4- and 5-gene structures.

We designed SIRA schemes to assemble three, four or five DNA fragments (‘cassettes’) into a plasmid vector in a single recombination reaction (Figure 2B). Each cassette is flanked by either two *attP* sites or two *attB* sites, except in the four-gene strategy, where one cassette has one *attP* and one *attB*. The central overlap sequences of the *att* sites are chosen such that each site can recombine with only one other type of site in the mixture. Recombination between all matching *attP*–*attB* pairs joins the cassettes together end-to-end in a specific order and inserts the resulting linear assembly into the pSIRA vector, replacing *ccdB* (Figure 2B).

To test these SIRA schemes, we assembled functional metabolic pathways from multiple genes. The carotenoid biosynthetic pathway of *Pantoea ananas* [*E. uredovora*] produces zeaxanthin (yellow) via lycopene (red) and β-carotene (yellow–orange) (27). Three genes from *P. ananas* (*crtE*, *crtB* and *crtI*) are sufficient to produce lycopene in *E. coli*, four genes (*crtE*, *crtB*, *crtI* and *crtY*) produce β-carotene and five genes (*crtE*, *crtB*, *crtI*, *crtY* and *crtZ*) produce zeaxanthin (Figure 2A).

The five carotenoid biosynthetic open reading frames were each amplified by PCR, with designed RBS upstream of their ATG start codons and terminal *attP* or *attB* incorporated in the PCR primers. The combinations of DNA fragments required for three-, four- or five-gene assemblies were mixed together with pSIRA1. Following incubation with ϕC31 integrase, the reactions were electroporated into *E. coli* TOP10*.* Plasmids that had lost the lethal *ccdB* gene, and therefore should contain the desired pathway, were selected by growth of transformants on medium containing ampicillin. The efficiency of assembly was measured as the percentage of coloured colonies obtained. As the number of fragments in the assembly was increased from three to five, both the number of colonies obtained and the efficiency of assembly decreased (Table 2; Figure 2). This decrease in efficiency was overcome by increasing the incubation time and adding ethylene glycol to the assembly reaction (Table 2; Supplementary Figure S3). A 22-h three-gene assembly reaction in the presence of 5% ethylene glycol yielded 1 × 10^6^ colonies (7 × 10^6^ colonies/ µg vector DNA), nearly 90% of which produced lycopene (red colonies) and contained plasmid with the expected structure, as confirmed by restriction analysis and DNA sequencing of plasmid DNA. Four- and five-gene assembly reactions gave nearly as many colonies (1 × 10^6^ and 7 × 10^5^, respectively), of which 18–37% produced the predicted coloured carotenoids and contained plasmids with the expected structure (Table 2; Figure 2B). The majority of incorrectly assembled plasmids contained a single gene from just one end of the desired assembly. These can be explained by recombination between one *attB* on pSIRA1 and a matching *attP* from the first or last cassette in the assembly, and mismatched recombination between the *attP* site at the other end of the cassette with the second *attB* from the vector. Although not a major problem for assembly reactions with pSIRA1, recombination between mismatched sites probably accounts for the poor efficiency of assembly reactions using pSIRA3, where intramolecular recombination between mismatched *attB^TT^* and *attP^TC^* sites can lead directly to deletion of *ccdB* (Supplementary Figures S3).

### Substituting and adding genes at specific positions within the assembly

**Figure 3.**
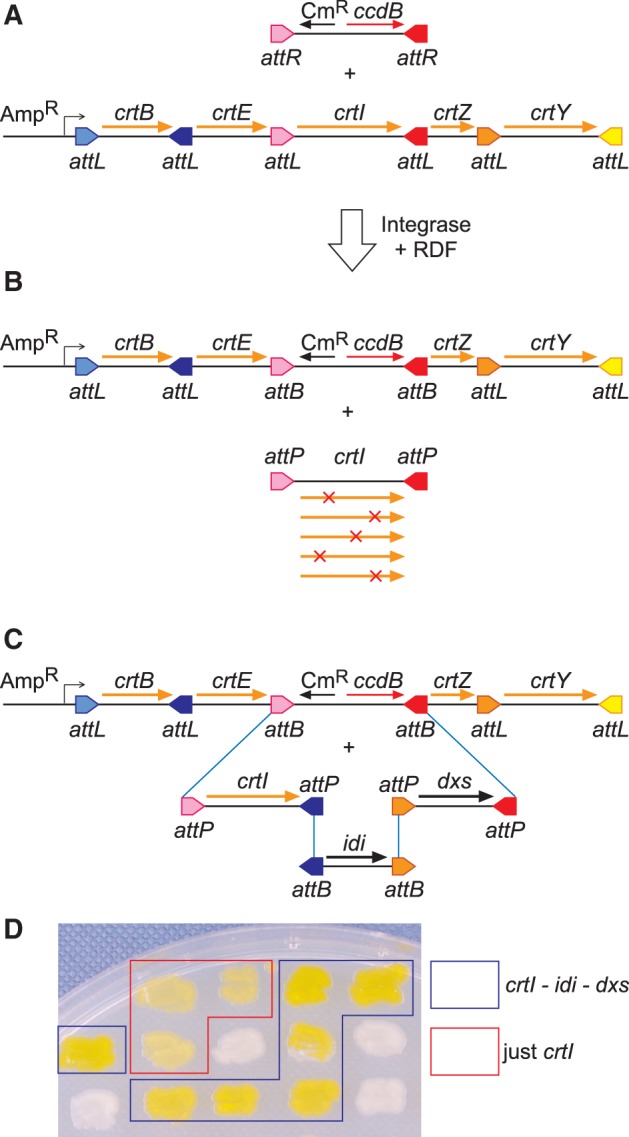
Replacement of a single gene in an assembly with ≥1 genes. (**A**) A *ccdB*-Cm^R^ cassette flanked by *attR* sites matching the gene to be replaced is produced by PCR. The chosen gene is then replaced using *attL* × *attR* recombination. (**B**) The product of this reaction contains two *attB* sites, and can be selected by chloramphenicol resistance in a *ccdB*-tolerant *E. coli* strain. The two *attB* sites can then recombine with matching *attP* sites to carry out a replacement reaction. (**C**) The two *attB* sites can be used to add multiple DNA cassettes using conventional SIRA assembly strategies. In both cases, the desired reaction products are selected by loss of the lethal *ccdB* gene. (**D**) A random selection of transformants from the reaction shown in (C) was patched onto solid media to assay carotenoid production. Approximately 50% of colonies contained the expected *idi*, *dxs* and *crtI* genes between *crtE* and *crtZ* and were more intensely coloured than those containing *crtI* alone.

The SIRA strategies described earlier in the text produce arrays of gene cassettes separated by *attL* sites, each with a different overlap sequence (Figure 3A). In the presence of integrase and RDF (ϕC31 Gp3) (24), each *attL* will recombine with a matching *attR* site. This recombination can potentially be used to replace any chosen DNA segment in the array. In practice, we found that direct replacement of a gene in the array by *attL* × *attR* recombination was less efficient than the original *attP* × *attB* SIRA reactions. We have observed DNA cleavage at *attL* sites in assemblies in the presence of integrase and RDF, presumably due to abortive recombination between mismatched *attL* sites in the array, which could cause these reduced efficiencies. To overcome this problem, we developed a two-step replacement strategy. A chosen segment of the assembly is first replaced with a selectable *ccdB*-Cm^R^ cassette, using *attL* × *attR* recombination. The *ccdB*-Cm^R^ cassette, now flanked by *attB* sites, can then be replaced with one or more DNA cassettes in an *attP* × *attB* SIRA reaction with integrase alone. Under these conditions, the remaining *attL* sites in the array are essentially inert and do not interfere with the reaction.

To illustrate how this procedure can be used to replace one gene with a library of gene variants, we replaced the wild-type *crtI* gene in the *crtBEIZY* zeaxanthin biosynthetic pathway with a mutagenized library of *crtI* genes. The *crtI* gene was first replaced with the *ccdB*-Cm^R^ cassette flanked by appropriate *attR* sites (Figure 3A), using ϕC31 integrase and RDF. Selection for chloramphenicol-resistant colonies in a *ccdB*-tolerant *E. coli* strain yielded hundreds of colonies. Ten of ten transformants examined carried the expected plasmid with *crtI* replaced by the *ccdB*-Cm^R^ cassette flanked by *attB* (Figure 3B). The *ccdB*-Cm^R^ cassette was then replaced by a Phusion- or Taq-polymerase amplified *crtI* cassette carrying appropriate *attP* sites in a standard SIRA reaction (Figure 3B), yielding >10^5^ colonies for both Phusion and Taq (Table 2). In all, 94% of colonies obtained with the high-fidelity Phusion polymerase had a functional zeaxanthin biosynthetic pathway as detected by production of yellow-coloured colonies, whereas only 65% of colonies from the mutagenic Taq reaction were coloured. DNA sequencing revealed that the colourless colonies contained plasmids with the expected (*crtBEIZY*) structure, but with mutations in *crtI* consistent with error-prone PCR.

The *attB* sites produced by replacing one gene in an assembly with the *ccdB*-Cm^R^ cassette could also be used to incorporate a further three, four, or five genes, yielding seven-, eight- or nine-gene assemblies (Figure 3C). To demonstrate this, we took the zeaxanthin metabolic pathway in which *crtI* had already been replaced with the *ccdB*-Cm^R^ cassette (Figure 3B) and added three linear PCR products, containing the *E. coli idi* and *dxs* genes and a new copy of *crtI* (Figure 3C). The *idi* and *dxs* genes are required for the synthesis of isopentenyl pyrophosphate (IPP) and dimethylallyl pyrophosphate (DMAPP), precursors in the synthesis of carotenoids (Figure 2). Overexpression of these enzymes in *E. coli* is reported to increase the available pools of these intermediates and thus increase carotenoid yields (28–31). SIRA reactions with ϕC31 integrase efficiently assembled these three genes into the zeaxanthin biosynthetic pathway, yielding plasmids with eight genes in the expected order, with an insert size of 7.8 kb (Figure 3C; Table 2). *E. coli* cells containing the full eight-gene assembly grew more vigorously than those containing just *crtBEIZY*, and were more intensely coloured, suggesting an increased yield of zeaxanthin (Figure 3D).

### Varying the order of genes in the assembly

**Figure 4.**
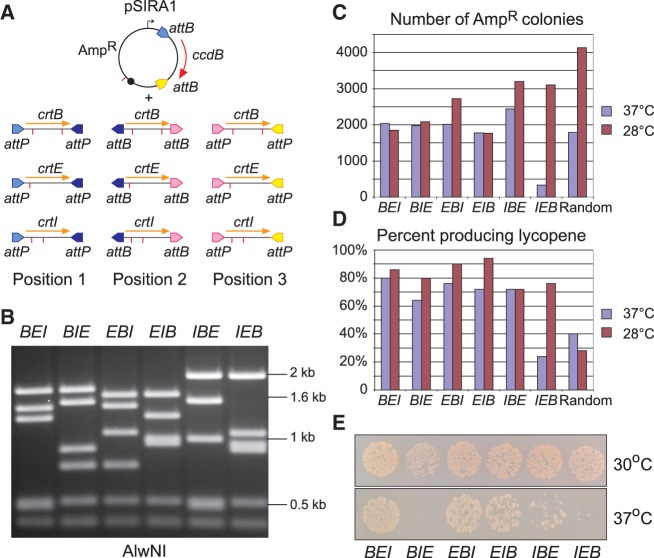
Assembly of lycopene biosynthetic operon in all possible gene orders. (**A**) The three genes for lycopene biosynthesis were assembled in all possible gene orders in pSIRA1. Each gene (*crtB*, *crtE* and *crtI*) was amplified by PCR with primers containing *att* sites specifying position 1 (*attP*^TT^ and *attP*^CT^), position 2 (*attB*^CT^ and *attB*^GT^) or position 3 (*attP*^GT^ and *attP*^TC^). By picking one gene for each position, all gene orders were assembled. Alternatively, by mixing all nine fragments in a single reaction, all possible gene orders are produced. The positions of AlwNI sites are indicated by red lines. (**B**) AlwNI restriction digests of representative plasmids containing *crtB*, *crtE* and *crtI* in each possible gene order. Gene orders are indicated above each lane as *BEI*, *BIE*, *EBI*, *EIB*, *IBE* and *IEB*. **(C)** Assembly reactions were set up in integrase reaction buffer with 5% ethylene glycol and 200 nM ϕC31 integrase and were incubated at 30°C for 24 h. Samples of each reaction mixture (1 µl) were used to transform chemically competent TOP10 cells, spread on LB-agar plates containing ampicillin and grown for 16 h at 37°C or 40 h at 28°C. The histogram shows the total number of colonies obtained for each transformation. (**D**) Randomly chosen transformant colonies were streaked on plates containing ampicillin and grown to assay for lycopene production. The histogram shows the percentage of colonies producing lycopene. (**E**) Overnight cultures of TOP10 *E. coli* carrying plasmids with *crtB*, *crtE* and *crtI* in each possible gene order were diluted 10^4^-fold in LB broth, plated in 10 µl spots and incubated overnight at 30°C and 37°C to display their colony formation and lycopene production phenotypes.

The assemblies described here have an artificial operon structure, with all genes in the same orientation, transcribed from a single upstream promoter. In this structure, genes closest to the promoter will typically be expressed at a higher level than those further away (32). Therefore, it should be possible to adjust the expression levels of the individual genes in an assembly by changing the order of genes in the operon. To illustrate this we used SIRA to assemble the three-gene lycopene biosynthetic pathway in all possible gene orders. Versions of each lycopene biosynthetic gene (*crtB*, *crtE* and *crtI*) were produced by PCR, with different terminal *att* sites to specify position one, position two or position three in a three-gene assembly (Figure 4A). By reacting one *crtB* fragment, one *crtE* fragment and one *crtI* fragment with pSIRA1, we easily assembled all six possible orders of these three genes (Figure 4B). Overexpression of the lycopene biosynthetic pathway has been reported to be toxic to *E. coli*, possibly because lycopene production diverts intermediates from essential metabolic pathways (33). Carotenoid yield and toxicity have also been reported to vary with temperature, with optimal lycopene production and lowest toxicity at 28–30°C (33–35) Assembly reactions for each gene order were transformed into *E. coli* TOP10, and transformants were incubated at either 28°C or 37°C. Assembly reactions for five of the six possible gene orders transformed *E. coli* efficiently at 37°C (Figure 4C), and 60–80% of transformant colonies were red (Figure 4D). One gene order (*crtIEB*) gave few colonies and a much lower frequency of red colonies when transformants were selected at 37°C (Figure 4C; D) suggesting that this gene order is toxic to *E. coli* at 37°C. To confirm this, the plating efficiency of cells containing each gene order in pSIRA1 was measured at 30 and 37°C. Gene orders *crtBIE* and *crtIEB* plated poorly at 37°C and gave noticeably smaller colonies than the other gene orders at 30°C (Figure 4E). There were also clear differences in lycopene production levels for the different gene orders; for example, *crtBEI* gave noticeably paler colonies than *crtEBI* at 30°C. Thus, changing the order of lycopene biosynthetic genes in pSIRA1 had clear phenotypic effects, and varying gene order could be a useful way to optimize production from biosynthetic pathways.

To select an optimal gene order, it might be advantageous to produce a library containing all possible gene orders. To do this, we reacted all nine *crt* gene cassettes (as described earlier in the text) with pSIRA1 in a single reaction. This reaction should produce assemblies of three genes in pSIRA1, with any one of the three *crt* genes at each position (27 combinations in total, of which 6 combinations will contain one copy of each gene; Figure 4A). When the reaction products were transformed into *E. coli*, ∼28% of transformants formed red colonies at 28°C, roughly as expected (6/27 = 22%) (Figure 4D). We verified that plasmids isolated from red colonies contained all three lycopene genes in all possible orders, whereas plasmids from white colonies contained other combinations of three cassettes (e.g. *crtB-crtB-crtI*; data not shown).

### Optimization of biosynthetic pathways by assembly with degenerate RBS

Another way to optimize expression levels of the different enzymes in a metabolic pathway is to vary the RBS of the genes in the pathway. Because we had observed toxic effects with lycopene overproduction, we used a different pathway to illustrate this approach. The violacein biosynthetic pathway from the Gram-negative bacterium *C. violaceum* produces a purple compound derived from tryptophan in a pathway requiring the activity of five enzymes encoded by *vioA*, *vioB*, *vioC*, *vioD* and *vioE* (36,37). These genes range in size from 576 to 2997 bp, and the total pathway is encoded on ∼7.5 kb of DNA. A prototype violacein biosynthetic pathway was built by amplifying the five *vio* genes directly from *C. violaceum* genomic DNA, using PCR primers incorporating designed RBS sequences and the required *attP* or *attB* sites. The resulting gene cassettes were assembled directly into pSIRA1 using ϕC31 integrase, yielding pale purple colonies (18% of all colonies; Table 2) containing the expected plasmid with all five *vio* genes. Starting from PCR primers and *C. violaceum* genomic DNA, it took just 2 days to obtain the correctly assembled functional pathway.

**Figure 5.**
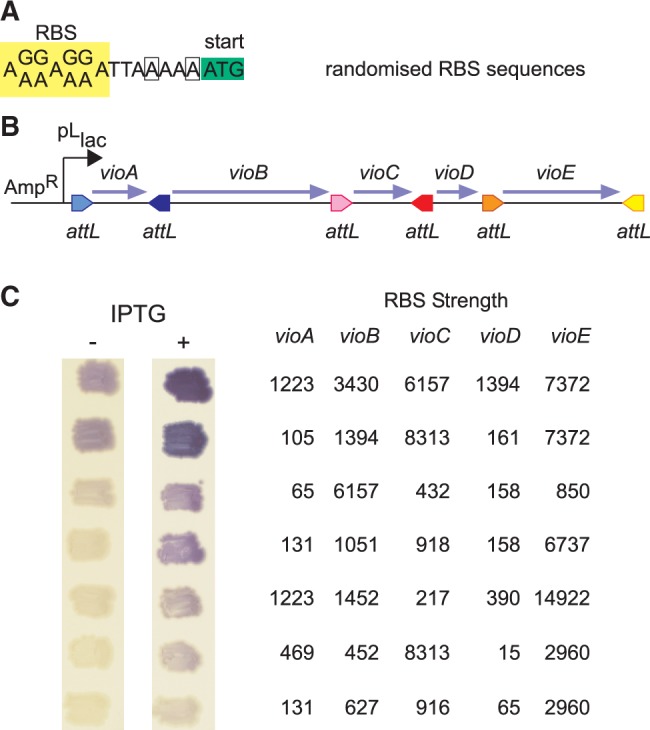
Assembly of the violacein biosynthetic pathway with degenerate RBS sequences. (**A**) Degenerate RBS sequence used for each gene in the violacein biosynthetic pathway. To obtain an appropriate range of predicted RBS strengths (37), the RBS sequences for *vioB* and *vioD* had AAAAT and ACAAA, respectively (changed sequences are shown boxed), rather than AAAAA upstream of the ATG. (**B**) Structure of the assembled synthetic *vio* operon, transcribed from an IPTG- regulated pL-lacO promoter in pSIRA5. (**C**) Phenotype of colonies containing *vio* assemblies with different RBS sequences at each gene, in the absence or presence of 0.1 mM IPTG. The table shows the predicted RBS strengths (arbitrary units) (37) for each gene in the operon.

Each of the *vio* genes was then amplified with primers incorporating degenerate RBS sequences (Figure 5A). The primers were designed to give 16 different RBS sequences for each gene, with predicted translation rates ranging from ∼100 to 10 000 units according to the RBS calculator software of Salis *et al.* (38). The PCR products were assembled into pSIRA5 [in which transcription is regulated by isopropylthio-β-galactoside (IPTG)] and electroporated into *ccdB*-sensitive *E. coli*. Colonies ranging in colour from light to intense purple were obtained on plates containing IPTG (Figure 5C). All colonies contained plasmids of the expected structure (data not shown) and violacein production was IPTG-inducible (Figure 5C). The RBS sequences were determined from seven representative plasmids and were shown to have different sequences consistent with the degenerate primers used. The predicted RBS strength was calculated for all genes in these seven pathways. The results suggest that *vioA* need not be highly expressed for high levels of violacein production, whereas low levels of *vioD* expression can be limiting (Figure 5C).

## DISCUSSION

We have shown here that ϕC31 integrase can be used to rapidly insert one, three, four or five genes or other DNA fragments in any chosen arrangement directly into a SIRA plasmid vector. We note that other serine integrases could substitute for ϕC31 integrase in all of the DNA manipulations shown here. Use of ≥2 integrases would allow the introduction of further orthogonal *attP*/*attB* pairs and would potentially allow the assembly of larger numbers of DNA segments in a single reaction.

The speed with which assemblies can be designed and produced by SIRA allows for rapid prototyping of metabolic pathways. Unlike other assembly methods, there is no need to subclone fragments into plasmid vectors before assembly or to remove internal restriction sites from within the DNA fragments. Assemblies can be easily modified by substituting ≥1 DNA segments with natural or artificial variants or by adding regulatory sequences or more genes at chosen locations.

The efficiency of SIRA facilitates the production of large combinatorial libraries with multiple variants of each DNA cassette at each position. One-fragment SIRA reactions routinely yield 8 × 10^7^ transformants per µg of vector DNA by electroporation (Table 2), higher than reported for homology-based cloning strategies such as Gibson assembly (7), SLiCE (8) and SLIC (6). Furthermore, the efficiency of SIRA holds up well compared with these methods as the number of DNA fragments is increased (Table 2).

The *attL* sites flanking the components of SIRA assemblies, each with different overlap sequences, could be used to move the complete assembly, or sub-assemblies, from one location to another. Site-specific recombination could shuttle assemblies between vectors with different antibiotic resistances, host ranges, or regulated promoters, or deliver metabolic pathways and devices to genomic ‘landing pads’ in chassis organisms.

Site-specific recombination has been used previously in a number of different approaches to insert DNA fragments into plasmid cloning vectors (39–43). However, to the best of our knowledge, this study is the first time that site-specific recombination has been used to assemble multiple genes into fully functional metabolic pathways. SIRA could easily be adapted for the assembly of synthetic genetic circuits or to generate translational fusions from multiple gene segments (e.g. to produce novel polyketide synthases from multiple enzymatic modules). Furthermore, SIRA is a highly flexible technology, which should be adaptable to many other applications in metabolic engineering and synthetic biology.

## SUPPLEMENTARY DATA

Supplementary Data are available at NAR Online.

## FUNDING

UK Engineering and Physical Sciences Research Council [EP/H019154/1 to S.J.R.]; the UK Biotechnology and Biological Sciences Research Council [BB/K003356/1 to W.M.S., BB/H005277/1 to M.C.M.S. and BB/J004561/1 to A.O.]; the National Science Foundation [0943392 to J.K.]; and the John Innes Foundation (to A.O.). Funding for open access charge: University of Glasgow.

*Conflict of interest statement*. None declared.

## Supplementary Material

Supplementary Data
