# Supplementary material for: Rapid metabolic pathway assembly and modification using serine integrase site-specific recombination
Source: Nucleic Acids Res. 2013 Nov 12;42(4):e23. doi: 10.1093/nar/gkt1101 (PMC3936721; doi:10.1093/nar/gkt1101)
Supplement: Supplementary Data [file supp_42_4_e23__index.html]

Rapid metabolic pathway assembly and modification using serine integrase site-specific recombination — Rapid metabolic pathway assembly and modification using serine integrase site-specific recombination — Supplementary Data 

# Rapid metabolic pathway assembly and modification using serine integrase site-specific recombination

## Supplementary Data

files

**Files in this Data Supplement:**

- Supplementary Data - pdf file
