# Supplementary material for: Rapid metabolic pathway assembly and modification using serine integrase site-specific recombination
Source: Nucleic Acids Res. 2013 Nov 12;42(4):e23. doi: 10.1093/nar/gkt1101 (PMC3936721; doi:10.1093/nar/gkt1101)
Supplement: Supplementary Data [file supp_gkt1101_nar-02888-met-h-2013-File007.pdf]

# Serine Integrase Directional Recombination (SIDR) for rapid metabolic pathway assembly and modification

## Supplementary Information

Sean D. Colloms[1]*, Christine A. Merrick[1], Femi J. Olorunniji[1], W. Marshall Stark[1], Maggie C. M. Smith[2], Anne Osbourn[3], Jay D. Keasling[4,5,6], and Susan J. Rosser[1]*

[1]Institute of Molecular, Cell and Systems Biology,
University of Glasgow,
Bower Building,
University Ave,
Glasgow G12 8QQ,
Scotland UK.

[2] Department of Biology, University of York, Wentworth Way, YO10 5DD, UK.

[3] Department of Metabolic Biology, The John Innes Centre, Norwich Research Park, Norwich NR4 7UH, UK.

[4] Joint BioEnergy Institute, 5885 Hollis Street, Emeryville, California 94608, USA.

[5]Department of Chemical and Biomolecular Engineering, University of California, Berkeley, CA 94720, USA

[6]Physical Biosciences Division, Lawrence Berkeley National Laboratory, Berkeley, CA 94720, USA

* Corresponding authors
Telephone: 0141-330-6236
email: Sean.Colloms@glasgow.ac.uk / Susan.Rosser@glasgow.ac.uk

## Supplementary Figures

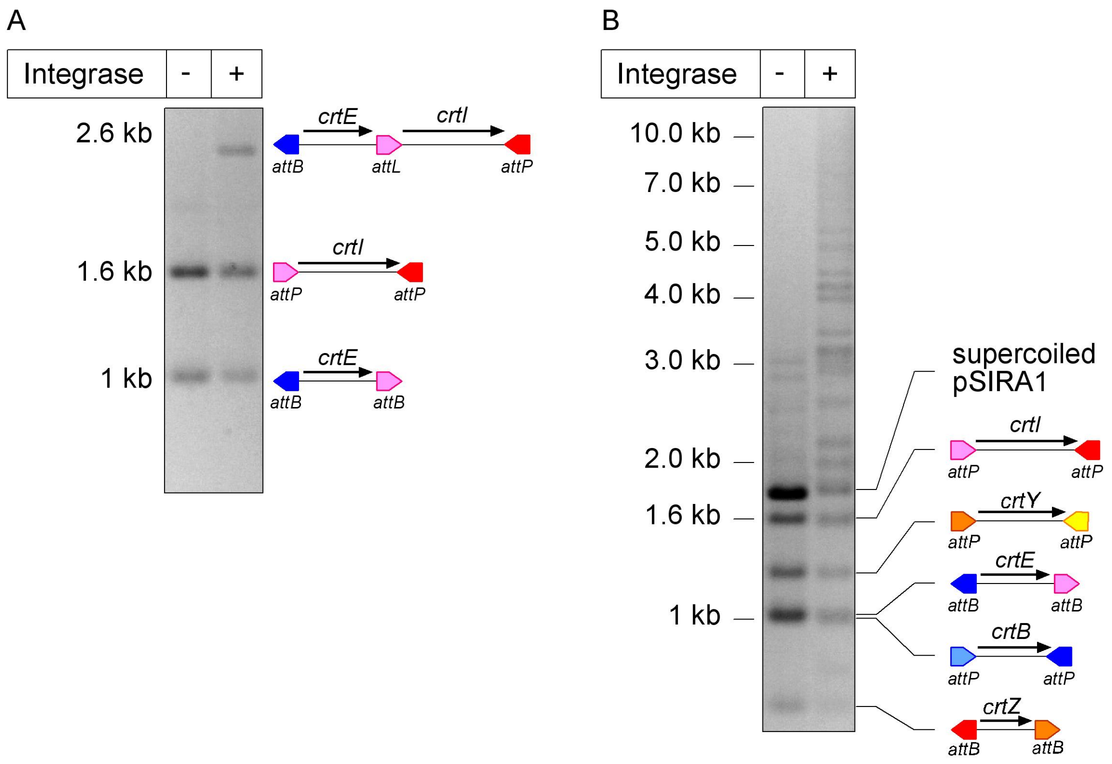


**Figure S1. (A)** Recombination specifically between fragments with matching *attP* and *attB* sites. A 1.0 kb *crtE* fragment with *attB*$^{CT}$ and *attB*$^{GT}$ sites and a 1.6 kb *crtI* fragment with *attP*$^{GT}$ and *attP*$^{CA}$ sites were incubated in the presence or absence of 200 nM ϕC31 integrase. The 2.6 kb product formed by recombination between *attB*$^{GT}$ and *attP*$^{GT}$ is indicated. **(B)** SIRA reaction to join the five zeaxanthin biosynthetic genes *crtB*, *crtE*, *crtI*, *crtY* and *crtZ* together and insert them into pSIRA1. Equimolar amounts of the indicated PCR products were incubated with pSIRA1 with or without 200nM integrase. All reactions contained ~5 nM of each PCR product and were incubated for four hours at 30°C in integrase reaction buffer with 10% ethylene glycol. Reactions were stopped by heating to 75°C for 10 minutes, treated with SDS and protease K and run on a 1.2% agarose Tris-Acetate EDTA (TAE) gel. The gel was stained with ethidium bromide and photographed on a Biorad GelDoc apparatus. Images are shown in reverse contrast.

**A**

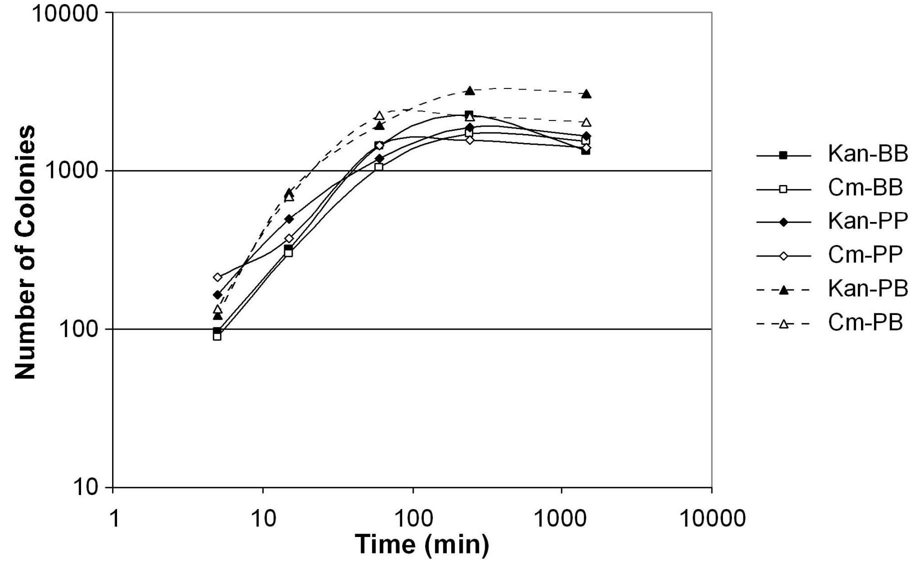


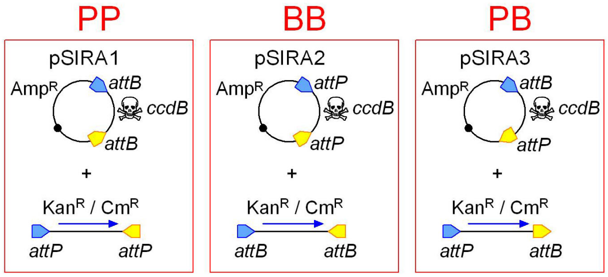


**Number of ampicillin resistant colonies**

| Time (min) | Kan-BB | Cm-BB | Kan-PP | Cm-PP | Kan-PB | Cm-PB |
|---|---|---|---|---|---|---|
| **No Integrase** | 0 | 0 | 0 | 0 | 0 | 0 |
| **5** | 97 | 89 | 164 | 212 | 122 | 134 |
| **15** | 320 | 298 | 496 | 374 | 734 | 694 |
| **60** | 1448 | 1060 | 1200 | 1448 | 1944 | 2260 |
| **240** | 2232 | 1712 | 1896 | 1560 | 3240 | 2224 |
| **1440** | 1344 | 1544 | 1672 | 1400 | 3096 | 2048 |

**Number of ampicillin resistant colonies (no insert)**

| Time (min) | pSIRA1 | pSIRA2 | pSIRA3 |
|---|---|---|---|
| **No Integrase** | 0 | 0 | 0 |
| **240** | 0 | 0 | 59 |
| **1440** | 0 | 0 | 68 |

**B**

**Fraction of colonies correctly assembled (resistant / total)**

| TIME | Kan-BB | Cm-BB | Kan-PP | Cm-PP | Kan-BP | Cm-BP |
|---|---|---|---|---|---|---|
| **5** | 20/20 | 20/20 | 20/20 | 20/20 | 22/22 | 19/20 |
| **15** | 20/20 | 21/21 | 20/20 | 20/20 | 20/20 | 20/20 |
| **60** | 20/20 | 22/22 | 20/20 | 20/20 | 20/20 | 22/22 |
| **240** | 20/20 | 20/20 | 20/20 | 20/20 | 64/66 | 64/66 |
| **1440** | 20/20 | 20/20 | 20/20 | 20/20 | 44/46 | 46/46 |

**Figure S2.** Time course of SIRA reactions to insert single linear DNA fragments into plasmid vectors. DNA fragments, containing a chloramphenicol resistance ($Cm^R$) or a kanamycin resistance ($Kan^R$) gene, were produced by PCR with pSW23 or pSW29 as template. The primers used incorporated *attB*$^{TT}$ upstream and *attB*$^{TC}$ downstream (BB), *attP*$^{TT}$ upstream and *attP*$^{TC}$ downstream (PP), or *attP*$^{TT}$ upstream and *attB*$^{TC}$ downstream (PB) of the resistance gene. Reactions (60 μl at 30°C) contained ~ 5 nM linear DNA fragment and ~5 nM pSIRA1 (PP), pSIRA2 (BB) or pSIRA3 (PB), and were set up in integrase reaction buffer without ethylene glycol. Recombination was initiated by the addition of 6 μl of 2 μM ϕC31 integrase. Samples (10 μl) were withdrawn after 5 minutes, 15 minutes, one hour, 4 hours and 24 hours and heated at 75°C for 10 minutes to inactivate the recombinase. A 2 μl aliquot from each time-point was transformed into chemically competent TOP10 cells (6 x $10^6$ tranformants / μg pUC19 DNA) and transformants were selected on LB-agar plates containing ampicillin. Control reactions were carried out with no integrase. **(A)** The number of ampicillin-resistant colonies obtained for each reaction are plotted against time in the graph and shown in the tables below. **(B)** Randomly chosen transformant colonies were streaked onto one plate containing ampicillin and another plate containing chloramphenicol or kanamycin as appropriate. The table shows the fraction of colonies expressing the expected antibiotic resistance gene (number of chloramphenicol- or kanamycin-resistant colonies / number of ampicillin-resistant colonies).

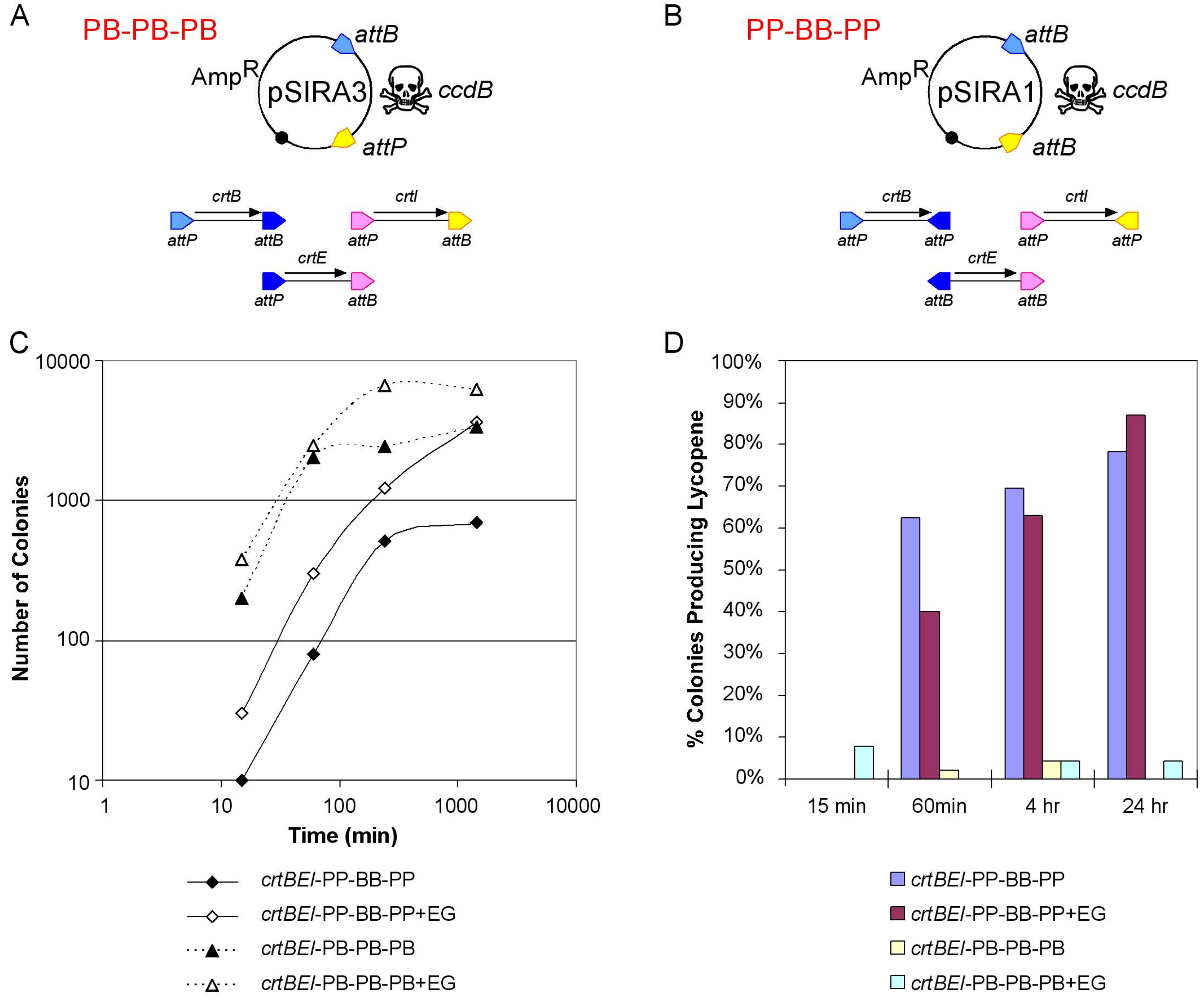


**Figure S3.** Time course of three-fragment SIRA reactions to assemble the *crtB-crtE-crtI* lycopene biosynthetic pathway. Assembly reactions were carried out with **(A)** pSIRA3 and linear PCR products, each with an upstream *attP* and a downstream *attB* (PB-PB-PB) or **(B)** with pSIRA1 and linear PCR products, each with either two *attP* or two *attB* sites (PP-BB-PP). Reactions (60 µl at 30°C) contained 5 nM of each linear DNA fragment and 3 nM SIRA vector and were in integrase reaction buffer with or without 5% ethylene glycol (EG). Recombination was initiated by the addition of 6 µl of 2 µM ϕC31 integrase. Samples (10 µl) were withdrawn after 15 minutes, one hour, 4 hours and 24 hours and heated at 75°C for 10 minutes to stop the reaction. A 2 µl aliquot from each time-point was transformed into chemically competent TOP10 cells ($10^6$ transformants / µg pUC19) and transformants were selected on LB-agar plates containing ampicillin. **(C)** Graph showing the number of ampicillin-resistant transformants obtained, plotted against reaction time. **(D)** Randomly chosen transformant colonies were streaked on plates containing ampicillin and grown overnight at 37°C. The histogram shows the percentage of colonies that were correctly assembled and therefore produced lycopene.

## Table S1 Oligonucleotides.

### S1.1 Oligonucleotides used for plasmid construction

| | |
|---|---|
| attP-RX-TT-top | AATTCAGTGCCCCAACTGGGGTAACCTTTGAGTTCTCTCAGTTGGGGGCGTT |
| attP-RX-TT-bot | CTAGAACGCCCCCAACTGAGAGAACTCAAAGGTTACCCCAGTTGGGGCACTG |
| attB-RX-TT-top | AATTCGTGCGGGTGCCAGGGCGTGCCCTTGGGCTCCCCGGGCGCGTACTCCT |
| attB-RX-TT-bot | CTAGAGGAGTACGCGCCCGGGGAGCCCAAGGGCACGCCCTGGCACCCGCACG |
| attP-PS-TC-top | GAGTGCCCCAACTGGGGTAACCTTCGAGTTCTCTCAGTTGGGGGCGTA |
| attP-PS-TC-bot | CTAGTACGCCCCCAACTGAGAGAACTCGAAGGTTACCCCAGTTGGGGCACTCTG CA |
| attB-PS-TC-top | GGTGCGGGTGCCAGGGCGTGCCCTCGGGCTCCCCGGGCGCGTACTCCA |
| attB-PS-TC-bot | CTAGTGGAGTACGCGCCCGGGGAGCCCGAGGGCACGCCCTGGCACCCGCACCT GCA |
| attP-SP-TC-top | CTAGAGTGCCCCAACTGGGGTAACCTTCGAGTTCTCTCAGTTGGGGGCGTCTGC A |
| attP-SP-TC-bot | GACGCCCCCAACTGAGAGAACTCGAAGGTTACCCCAGTTGGGGCACT |
| pL-Eco-top | ACTGACCAAGCTTGCTCTAGA |
| pL-Eco-bot | AATTTCTAGAGCAAGCTTGGTCAGTGCGTCCTGCTGATGTGCTCA |
| pL-tet-top | AATTCTTAAGTCCCTATCAGTGATAGAGATTGACATCCCTATCAGTGATAGAGATA CTGAGCACATCAGCAGGACGC |
| pL-tet-bot | GTATCTCTATCACTGATAGGGATGTCAATCTCTATCACTGATAGGGACTTAAG |
| pL-lac-top | AATTCTTAAGAATTGTGAGCGGATAACAATTGACATTGTGAGCGGATAACAAGATA CTGAGCACATCAGCAGGACGC |
| pL-lac-bot | GTATCTTGTTATCCGCTCACAATGTCAATTGTTATCCGCTCACAATTCTTAAG |

## Table S1.2 PCR primers for initial isolation of *crt* genes

| | |
|---|---|
| *crtB*-EU-for | **AGGAGGA**TTACAAA**ATG**AATAATCCGTCGTTACTCAATCATGCGG |
| *crtB*-EU-rev | **TTA**GAGCGGGCGCTGCCAG |
| *crtE*-EU-for | **AGGAGG**ATTACAAA**ATG**ACGGTCTGCGCAAAAAAACACG |
| *crtE*-EU-rev | **TTA**ACTGACGGCAGCGAGTTTTTTGTC |
| *crtI*-EU-for | **AGGAGGA**TTACAAA**ATG**AAACCAACTACGGTAATTGGTGCAGG |
| *crtI*-EU-rev | **TTA**TATCAGATCCTCCAGCATCAAACCTGC |
| crtY-EU-for | **AGGAGGA**TTACAAA**ATG**CAACCGCATTATGATCTGATTCTCG |
| crtY-EU-rev | **TTA**ACGATGAGTCGTCATAATGGCTTGC |

## Table S1.3 PCR primers incorporating *attP* and *attB* sequences

| | |
|---|---|
| attB-TT-antibiotic-resistance-F | GAATTCGTGCGGGTGCCAGGGCGTGCCCTTGGCTCCCCGGGCGCGTACTCCA AGAGGTTCCAACTTTCACCAT |
| attB-TC-antibiotic-resistance-R | CCATGGTGCGGGTGCCAGGGCGTGCCCTCGGCTCCCCGGGCGCGTACTCCTT TCTAGGCACCAATAACTGC |
| attP-TT-antibiotic-resistance-F | TCTAGAAGTGCCCCAACTGGGGTAACCTTTGAGTTCTCTCAGTTGGGGGCGTAAG AGGTTCCAACTTTCACCAT |
| attP-TC-antibiotic-resistance-R | AGCACTAGTAGTGCCCCAACTGGGGTAACCTTCGAGTTCTCTCAGTTGGGGGCG TTTTCTAGGCACCAATAACTGC |
| attB-TC-antibiotic-resistance-R | ACTAGTGGAGTACGCGCCCGGGGAGCCCGAGGGCACGCCCTGGCACCCGCACT TTCTAGGCACCAATAACTGC |
| attP-TT-crtB-F | AGCTCTAGAAGTGCCCCAACTGGGGTAACCTTTGAGTTCTCTCAGTTGGGGGCGT **AGGAGGA**TTACAAA**ATG**AATAATCCGTCG |
| attP-CT-crtB-R | AGCAAATTCAGTGCCCCAACTGGGGTAACCTCTGAGTTCTCTCAGTTGGGGGCGT **TTA**GAGCGGGCGCTGCCAG |
| attB-CT-crtB-R | AGCACTAGTGGAGTACGCGCCCGGGGAGCCCAGGGGCACGCCCTGGCACCCG CAC**TTA**GAGCGGGCGCTGCCAG |
| attB-CT-crtE-F | AGCTCTAGAGGAGTACGCGCCCGGGGAGCCCAGGGGCACGCCCTGGCACCCG CAC**AGGAGGA**TTACAAA**ATG**ACGGTCTGC |
| attP-CT-crtE-F | AGCTCTAGAAGTGCCCCAACTGGGGTAACCTCTGAGTTCTCTCAGTTGGGGGCG T**AGGAGGA**TTACAAA**ATG**ACGGTCTGC |
| attB-GT-crtE-R | AGCACTAGTGGAGTACGCGCCCGGGGAGCCCACGGGCACGCCCTGGCACCCGC AC**TTA**ACTGACGGCAGCGAGTTTTTTGTC |

| | |
|---|---|
| attP-GT-crtI-F | AGCTCTAGAAGTGCCCCAACTGGGGTAACCTGTGAGTTCTCTCAGTTGGGGGCG T**AGGAGGA**TTACAAA**ATG**AAACCAACTACG |
| attP-CT-crtI-R | AGCGAATTCAGTGCCCCAACTGGGGTAACCTCTGAGTTCTCTCAGTTGGGGGCG T**TTA**TATCAGATCCTCCAGCATCAAACCTGC |
| attP-CA-crtI-R | AGCACTAGTAGTGCCCCAACTGGGGTAACCTCAGAGTTCTCTCAGTTGGGGGCG T**TTA**TATCAGATCCTCCAGCATCAAACCTGC |
| attP-TC-crtI-R | AGCACTAGTAGTGCCCCAACTGGGGTAACCTTCGAGTTCTCTCAGTTGGGGGCG T**TTA**TATCAGATCCTCCAGCATCAAACCTGC |
| attB-CC-crtI-R | AGCACTAGTGGAGTACGCGCCCGGGGAGCCCGGGGGCACGCCCTGGCACCCG CAC**TTA**TATCAGATCCTCCAGCATCAAACCTGC |
| attB-TC-crtI-R | AGCACTAGTGGAGTACGCGCCCGGGGAGCCCGAGGGCACGCCCTGGCACCCG CAC**TTA**TATCAGATCCTCCAGCATCAAACCTGC |
| attP-CC-crtY-F | AGCTCTAGAAGTGCCCCAACTGGGGTAACCTCCGAGTTCTCTCAGTTGGGGGCG T**AGGAGG**ATTACAAA**ATG**CAACCGC |
| attP-TC-crtY-R | AGCACTAGTAGTGCCCCAACTGGGGTAACCTTCGAGTTCTCTCAGTTGGGGGCG T**TTA**ACGATGAGTCGTCATAATGGCTTGC |
| attB-CA-crtZ-F | AGCTCTAGAGGAGTACGCGCCCGGGGAGCCCTGGGGCACGCCCTGGCACCCGC AC**AGGAGGA**TTACATT**ATG**TTGTGGATTTGGAATGC |
| attB-CC-crtZ-R | AGCACTAGTGGAGTACGCGCCCGGGGAGCCCGGGGGCACGCCCTGGCACCCG CACCC**TTA**CTTCCCGGATGC |
| attP-TT-crt*-F | AGCACTAGTAGTGCCCCAACTGGGGTAACCTTTGAGTTCTCTCAGTTGGGGGCGT TCGCCCTTAGGAGGATTACA |
| attP-CT-crt*-R | AGCACTAGTAGTGCCCCAACTGGGGTAACCTCTGAGTTCTCTCAGTTGGGGGCG TGCTGGAATTCGCCCTTTTA |
| attB-CT-crt*-F | AGCTCTAGAGGAGTACGCGCCCGGGGAGCCCAGGGGCACGCCCTGGCACCCG CACTCGCCCTTAGGAGGATTACA |
| attB-GT-crt*-R | AGCTCTAGAGGAGTACGCGCCCGGGGAGCCCACGGGCACGCCCTGGCACCCGC ACGCTGGAATTCGCCCTTTTA |
| attP-GT-crt*-F | AGCACTAGTAGTGCCCCAACTGGGGTAACCTGTGAGTTCTCTCAGTTGGGGGCG TTCGCCCTTAGGAGGATTACA |
| attP-TC-crt*-R | AGCACTAGTAGTGCCCCAACTGGGGTAACCTTCGAGTTCTCTCAGTTGGGGGCG TGCTGGAATTCGCCCTTTTA |
| attR-GT-BB-F | AGCACTAGTAGTGCCCCAACTGGGGTAACCTGTGGGCTCCCCGGGCGCGTACTC CCGCTAAGGATGATTTCTGGA |
| attR-CA-BB-R | AGCACTAGTAGTGCCCCAACTGGGGTAACCTCAGGGCTCCCCGGGCGCGTACTC CGGTGACACCTTGCCCTTTT |
| attB-CT-idi-F | AGCTCTAGAGGAGTACGCGCCCGGGGAGCCCAGGGGCACGCCCTGGCACCCG CAC**AGGAGGA**TTACAAA**ATG**CAAACGGAACACGTCAT |
| attB-CC-idi-R | AGCACTAGTGGAGTACGCGCCCGGGGAGCCCGGGGGCACGCCCTGGCACCCG CAC**TTA**TTTAAGCTGGGTAAATGCAGATA |
| attP-CC-dxs-F | AGCTCTAGAAGTGCCCCAACTGGGGTAACCTCCGAGTTCTCTCAGTTGGGGGCG T**AGGAGGA**TTACCTG**ATG**AGTTTTGATATTGCC |
| attP-CA-dxs-R | GCACTAGTAGTGCCCCAACTGGGGTAACCTCAGAGTTCTCTCAGTTGGGGGCGT GGGA**TTA**TGCCAGCCAGG |

| | |
|---|---|
| attP-TT-vioA-F | AGCTCTAGAAGTGCCCCAACTGGGGTAACCTTTGAGTTCTCTCAGTTGGGGGCGT **AGGAGGA**TTACAAA**ATG**AAGCATTCTTCCGATATCTGC |
| attP-CT-vioA-R | AGCAATTCAGTGCCCCAACTGGGGTAACCTCTGAGTTCTCTCAGTTGGGGGCGT**T** **TA**CGCGGCGATGCGCTGCAGCAG |
| attB-CT-vioB-F | AGCTCTAGAGGAGTACGCGCCCGGGGAGCCCAGGGGCACGCCCTGGCACCCG CAC**AGGAGGA**TTACAAA**ATG**AGCATTCTGGATTTTCCACGC |
| attB-GT-vioB-R | AGCACTAGTGGAGTACGCGCCCGGGGAGCCCACGGGCACGCCCTGGCACCCGC AC**TTA**GGCCTCTCTAGAAAGCTTTCC |
| attP-GT-vioC-F | AGCTCTAGAAGTGCCCCAACTGGGGTAACCTGTGAGTTCTCTCAGTTGGGGGCG T**AGGAGGA**TTACAAA**ATG**AAAAGAGCAATCATAGTCGGA |
| attP-CA-vioC-R | GCACTAGTAGTGCCCCAACTGGGGTAACCTCAGAGTTCTCTCAGTTGGGGGCGT **TTA**GTTGACCCTCCCTATCTTGTAC |
| attB-CA-vioD-F | AGCTCTAGAGGAGTACGCGCCCGGGGAGCCCTGGGGCACGCCCTGGCACCCGC AC**AGGAGGA**TTACAAA**ATG**AAGATTCTGGTCATCGGCGCG |
| attB-CC-vioD-R | AGCACTAGTGGAGTACGCGCCCGGGGAGCCCGGGGGCACGCCCTGGCACCCG CAC**TTA**GCGTTGCAGCGCGTAGCGCAG |
| attP-CC-vioE-F | AGCTCTAGAAGTGCCCCAACTGGGGTAACCTCCGAGTTCTCTCAGTTGGGGGCG T**AGGAGGA**TTACAAA**ATG**GAAAACCGGGAACCGCCGCTG |
| attP-TC-vioE-R | AGCACTAGTAGTGCCCCAACTGGGGTAACCTTCGAGTTCTCTCAGTTGGGGGCG T**TTA**GCGCTTGGCGGCGAAGACGGC |
| D-attP-TT-vioA-F | AGCTCTAGAAGTGCCCCAACTGGGGTAACCTTTGAGTTCTCTCAGTTGGGGGCGT **ARRARRA**TTAAAAA**ATG**AAGCATTCTTCCGATATCTGC |
| D-attB-CT-vioB-F | AGCTCTAGAGGAGTACGCGCCCGGGGAGCCCAGGGGCACGCCCTGGCACCCG CAC**ARRARRA**TTAAAAT**ATG**AGCATTCTGGATTTTCCACGC |
| D-attP-GT-vioC-F | AGCTCTAGAAGTGCCCCAACTGGGGTAACCTGTGAGTTCTCTCAGTTGGGGGCG T**ARRARRA**TTAAAAAATGAAAAGAGCAATCATAGTCGGA |
| D-attB-CA-vioD-F | AGCTCTAGAGGAGTACGCGCCCGGGGAGCCCTGGGGCACGCCCTGGCACCCGC AC**ARRARRA**TTACAAAATGAAGATTCTGGTCATCGGCGCG |
| D-attP-CC-vioE-F | AGCTCTAGAAGTGCCCCAACTGGGGTAACCTCCGAGTTCTCTCAGTTGGGGGCG T**ARRARRA**TTAAAAA**ATG**GAAAACCGGGAACCGCCGCTG |

*attP* sequences are shown highlighted in yellow, *attB* sequences are highlighted in cyan. Bottom strand *attP* and *attB* sequences are underlined. Central dinucleotides (named according to their sequence on the top strand) are highlighted red. Ribosome binding sites (generally AGGAGGA) and start codons (ATG) in forward primers, and the reverse complement of stop codons (TTA) in reverse primers are shown underlined and bold.

**Table S1.4 Sequencing Primers**

| | |
|---|---|
| VF2 | TGCCACCTGACGTCTAAGAA |
| VR | ATTACCGCCTTTGAGTGAGC |
| crtB-out | CTGGAGCATGAAGGTCTGAA |
| crtE-out | GACGCTGGTCAATCTGTTAG |
| crtE-int-for | AACGTATTCGCCCCATGTT |
| crtI-int-for | GCCACCTCATCCATTTATAC |
| crtI-int-rev | CCACCCAGATCCTGAAACAG |
| crtI-3’-out | TCTGTGGAGCCCGTTCTTAC |
| crtI-out | GCATAACCGCGATAAAACCA |
| vioA-out | GTTCTGCCGCGACAGCGATA |
| vioB-out | ATACCCGCAGCTACGACGAC |
| vioC-out | ACACCCGCTACATGCATAGC |
| vioD-out-2 | AGTCCGGCCACTTCTCCAT |

## Table S2 Summary of PCRs for assembly reactions

| **1 gene (*attP attP*)** | **$Cm^R$ into pSIRA1** | |
|---|---|---|
| Forward Primer | Reverse Primer | Template |
| *attP*-TT-antibiotic-resistance-F | *attP*-TC-antibiotic-resistance-R | pSW23 |

| **1 gene (*attP attB*)** | **$Cm^R$ into pSIRA3** | |
|---|---|---|
| Forward Primer | Reverse Primer | Template |
| *attP*-TT-antibiotic-resistance-F | *attB*-TC-antibiotic-resistance-R | pSW23 |

| **1 gene (*attB attB*)** | **$Cm^R$ into pSIRA2** | |
|---|---|---|
| Forward Primer | Reverse Primer | Template |
| *attB*-TT-antibiotic-resistance-F | *attB*-TC-antibiotic-resistance-R | pSW23 |

| **1 gene (*attP attP*)** | **$Kan^R$ into pSIRA1** | |
|---|---|---|
| Forward Primer | Reverse Primer | Template |
| *attP*-TT-antibiotic-resistance-F | *attP*-TC-antibiotic-resistance-R | pSW29 |

| **1 gene (*attP attB*)** | **$Kan^R$ into pSIRA3** | |
|---|---|---|
| Forward Primer | Reverse Primer | Template |
| *attP*-TT-antibiotic-resistance-F | *attB*-TC-AbR | pSW29 |

| **1 gene (attB attB)** | **$Kan^R$ into pSIRA2** | |
|---|---|---|
| Forward Primer | Reverse Primer | Template |
| attB-TT-antibiotic-resistance-F | attB-TC-antibiotic-resistance-R | pSW29 |

| **3 genes** | ***crtB, crtE, crtI* into pSIRA1** | |
|---|---|---|
| Forward Primer | Reverse Primer | Template |
| attP-TT-crtB-F | attP-CT-crtB-R | pCM1 |
| attB-CT-crtE-F | attB-GT-crtE-R | pCM2 |
| attP-GT-crtI-F | attP-TC-crtI-R | pCM3 |

| **3 genes** | ***crtB, crtE, crtI* into pSIRA3** | |
|---|---|---|
| Forward Primer | Reverse Primer | Template |
| attP-TT-crtB-F | attB-CT-crtB-R | pCM1 |
| attP-CT-crtE-F | attB-GT-crtE-R | pCM2 |
| attP-GT-crtI-F | attB-TC-crtI-R | pCM3 |

| **4 genes** | ***crtB, crtE, crtI,  crtY* into pSIRA1** | |
|---|---|---|
| Forward Primer | Reverse Primer | Template |
| attP-TT-crtB-F | attP-CT-crtB-R | pCM1 |
| attB-CT-crtE-F | attB-GT-crtE-R | pCM2 |
| attP-GT-crtI-F | attB-CC-crtI-R | pCM3 |
| attP-CC-crtY-F | attP-TC-crtY-R | pCM4 |

| **5 genes** | ***crtB, crtE, crtI, crtZ, crtY* into pSIRA1** | |
|---|---|---|
| Forward Primer | Reverse Primer | Template |
| attP-TT-crtB-F | attP-CT-crtB-R | pCM1 |
| attB-CT-crtE-F | attB-GT-crtE-R | pCM2 |
| attP-GT-crtI-F | attP-CA-crtI-R | pCM3 |
| attB-CA-crtZ-F | attB-CC-crtZ-R | *P. agglomerans* genomic DNA |
| attP-CC-crtY-F | attP-TC-crtY-R | pCM4 |

| **3 genes random order** | ***crtB, crtE, crtI, crtZ, crtY* into pSIRA1** | |
|---|---|---|
| Forward Primer | Reverse Primer | Template |
| attP-TT-crt*-F | attP-CT-crt*-R | pCM1 |
| attB-CT-crt*-F | attB-GT-crt*-R | pCM1 |
| attP-GT-crt*-F | attP-TC-crt*-R | pCM1 |
| attP-TT-crt*-F | attP-CT-crt*-R | pCM2 |
| attB-CT-crt*-F | attB-GT-crt*-R | pCM2 |
| attP-GT-crt*-F | attP-TC-crt*-R | pCM2 |
| attP-TT-crt*-F | attP-CT-crt*-R | pCM3 |
| attP-CT-crt*-F | attB-GT-crt*-R | pCM3 |
| attP-GT-crt*-F | attP-TC-crt*-R | pCM3 |

| **5 genes** | ***vioA vioB, vioC, vioD, vioE* into pSIRA4** | |
|---|---|---|
| Forward Primer | Reverse Primer | Template |
| attP-TT-vioA-F | attP-CT-vioA-R | *C. violaceum* genomic DNA |
| attB-CT-vioB-F | attB-GT-vioB-R | *C. violaceum* genomic DNA |
| attP-GT-vioC-F | attP-CA-vioC-R | *C. violaceum* genomic DNA |
| attB-CA-vioD-F | attB-CC-vioD-R | *C. violaceum* genomic DNA |
| attP-CC-vioE-F | attP-TC-vioE-R | *C. violaceum* genomic DNA |

| **5 genes** | ***vioA vioB, vioC, vioD, vioE* degenerate RBS into pSIRA4** | |
|---|---|---|
| Forward Primer | Reverse Primer | Template |
| D-attP-TT-vioA-F | attP-CT-vioA-R | *C. violaceum* genomic DNA |
| D-attB-CT-vioB-F | attB-GT-vioB-R | *C. violaceum* genomic DNA |
| D-attP-GT-vioC-F | attP-CA-vioC-R | *C. violaceum* genomic DNA |
| D-attB-CA-vioD-F | attB-CC-vioD-R | *C. violaceum* genomic DNA |
| D-attP-CC-vioE-F | attP-TC-vioE-R | *C. violaceum* genomic DNA |

| ***ccdB*-Cm$^R$ cassette** | **into pSIRA1 *crtB-crtE-crtI-crtZ-crtY*** | |
|---|---|---|
| Forward Primer | Reverse Primer | Template |
| attR-GT-BB-F | attR-CA-BB-R | p-Cm$^R$-ccdB |

| ***crtI* mutant library** | **into pSIRA1 *crtB-crtE-ccdB-Cm$^R$-crtZ-crtY*** | |
|---|---|---|
| Forward Primer | Reverse Primer | Template |
| attP-GT-crtI-F | attP-CA-crtI-R | pCM3 |

| ***3 genes crtI-dxs-idi*** | **into pSIRA1 *crtB-crtE-ccdB-Cm$^R$-crtZ-crtY*** | |
|---|---|---|
| Forward Primer | Reverse Primer | Template |
| attP-GT-crtI-F | attP-CT-crtI-R | pCM3 |
| attB-CT-idi-F | attB-CC-idi-R | *E. coli* genomic DNA |
| attP-CC-dxs-F | attP-CA-dxs-R | *E. coli* genomic DNA |
